# Case Report: Neurosyphilis with a paraparetic Guillain-Barré-spectrum phenotype and CSF-detected anti-GT1a IgG: an infection-autoimmunity diagnostic challenge

**DOI:** 10.3389/fimmu.2026.1936622

**Published:** 2026-09-14

**Authors:** Xing Zhao, Yizhi Zhang, Wenjing Xie, Fei Yin

**Affiliations:** Department of Neurology, The Second Hospital of Jilin University, Changchun, China

**Keywords:** anti-GT1a antibody, blood-CSF barrier, case report, Guillain-Barré syndrome, neurosyphilis, polyradiculoneuropathy

## Abstract

**Background:**

Active central nervous system (CNS) infection can resemble acute immune polyradiculoneuropathy, and the two processes may coexist. We describe a patient with neurosyphilis, rapidly progressive paraparetic weakness, and anti-GT1a IgG detected in cerebrospinal fluid (CSF) but not serum by a qualitative line immunoblot.

**Case presentation:**

A 41-year-old man developed rapidly progressive paraparesis, urinary retention, and left abducens palsy. On hospital day 2, CSF opening pressure was 260 mmH_2_O, protein 1.56 g/L, white-cell count 272 × 10^6^/L, CSF TPPA was reactive, and CSF RPR was 1:8. Penicillin and methylprednisolone were started after the first lumbar puncture. By day 8, CSF white-cell count and protein had fallen during overlapping antimicrobial and corticosteroid treatment, but opening pressure remained 260 mmH_2_O and neurological deficits persisted. Paired serum-CSF testing showed marked blood-CSF barrier dysfunction (QAlb 17.4 × 10^-3^), a type IV oligoclonal-band pattern, a Reiber-derived intrathecal IgM fraction of 35%, and anti-GT1a IgG detected in CSF but not serum. Day-11 electrodiagnostic testing showed reduced lower-limb motor amplitudes, preserved sensory responses, and absent F waves; a stable GBS electrophysiological subtype could not be assigned. Initial spinal MRI was normal; repeat lumbar MRI about 20 days later showed smooth enhancement along the ventral conus and cauda equina roots. IVIG was given on days 11-15; improvement was documented by day 30, and at 2 years the patient reported return to normal life and work.

**Conclusion:**

Neurosyphilis can present with a paraparetic GBS-spectrum phenotype, making it difficult to determine whether syphilitic meningoradiculitis alone explains the syndrome or whether a concurrent immune polyradiculoneuropathy is also present. In this setting, blood-CSF barrier dysfunction, CSF-detected anti-GT1a IgG, and improvement after IVIG should be viewed as hypothesis-generating observations rather than proof of antibody compartmentalization, dual pathology, or treatment causality. In similar cases, longitudinal clinical follow-up, electrophysiology, imaging, and quantitatively validated paired serum-CSF immune testing are needed to distinguish a single infectious process, a concurrent immune neuropathy, and a biologically linked infection-autoimmunity process.

## 1 Introduction

Central nervous system (CNS) infection and acute immune neuropathy can look similar clinically. Neurosyphilis may involve the meninges, cranial nerves, spinal roots, and peripheral nerves and can therefore resemble Guillain-Barré syndrome (GBS) (2–5). Only a few reports have described GBS-like presentations in patients with neurosyphilis (2, 3, 8). Marked CSF pleocytosis is unusual in GBS and should prompt a search for infection or another diagnosis (7). Nerve-root enhancement and improvement after immunotherapy are also nonspecific in this setting.

We report a patient with active neurosyphilis, rapidly progressive paraparetic weakness, a motor-predominant lower-limb electrophysiological pattern on a single study, and anti-GT1a IgG detected in CSF but not serum by qualitative line immunoblot. The central question is whether neurosyphilis fully explains the acute peripheral-appearing syndrome. The findings permit three competing interpretations: syphilitic meningoradiculitis as a single-process explanation, an independent concurrent GBS-spectrum neuropathy, or a biologically linked infection-autoimmunity process. Because the anti-GT1a assay was qualitative and did not provide a validated quantitative comparison across serum and CSF matrices, the observed discordance cannot establish antibody enrichment, compartmental restriction, or intrathecal synthesis. We therefore separate the observed findings from mechanistic interpretation and use a non-causal, testable framework to compare these explanations.

## 2 Case description

A 41-year-old man presented after 5 days of weakness that began in the right leg and progressed to severe, approximately symmetric lower-limb weakness with inability to lift either leg. He reported urinary retention, numbness of both feet, posterior calves, and buttocks, and diplopia that worsened on left gaze. He denied dysphagia, a truncal sensory level, or band-like thoracoabdominal dysesthesia. His history included diabetes mellitus treated with insulin and hypothyroidism treated with levothyroxine. Penicillin treatment for syphilis in 2014 was patient-reported; contemporaneous records, disease stage, exact regimen, serological response, and CSF data were unavailable. He denied subsequent high-risk sexual exposure, but relapse or treatment failure could not be distinguished definitively from reinfection. He had no rash or lymphadenopathy.

Neurological examination showed limited left-eye abduction with gaze-evoked horizontal nystagmus. Strength was Medical Research Council (MRC) grade 1/5 in both legs and normal in the arms. Tone and tendon reflexes were reduced in the legs, whereas upper-limb reflexes were preserved. Sensory testing showed distal stocking-glove hypalgesia; proprioception was preserved; plantar responses were flexor bilaterally. No ataxia was documented. Fundoscopy showed no papilledema, and the patient did not report headache, nausea, vomiting, or other symptoms suggestive of raised intracranial pressure. Formal GBS disability and modified Rankin scores were not recorded. The isolated abducens palsy was not classified as Miller Fisher syndrome because the classic phenotype was incomplete and alternative explanations remained plausible (6).

Routine blood testing, including complete blood count, electrolytes, renal and liver panels, creatine kinase, vitamin B12/folate, antinuclear and antineutrophil cytoplasmic antibodies, was unrevealing; HIV testing was negative. Glycated hemoglobin was 5.5%. Serum TPPA was reactive and serum RPR was 1:32. A systematic antecedent-infection panel for GBS (for example, Campylobacter jejuni, cytomegalovirus, Epstein-Barr virus, or Mycoplasma pneumoniae) was not obtained. Brain MRI showed no parenchymal lesion but incidentally demonstrated cerebellar tonsillar ectopia. Initial spinal MRI was normal (**Figures 1A–D**); repeat contrast-enhanced lumbar MRI approximately 20 days later showed smooth linear enhancement along the ventral conus medullaris and cauda equina roots (**Figures 1E–H**). This finding was compatible with radiculitis but was not specific for autoimmune GBS and could also occur with infectious meningoradiculitis (14, 15).

**Figure 1:**
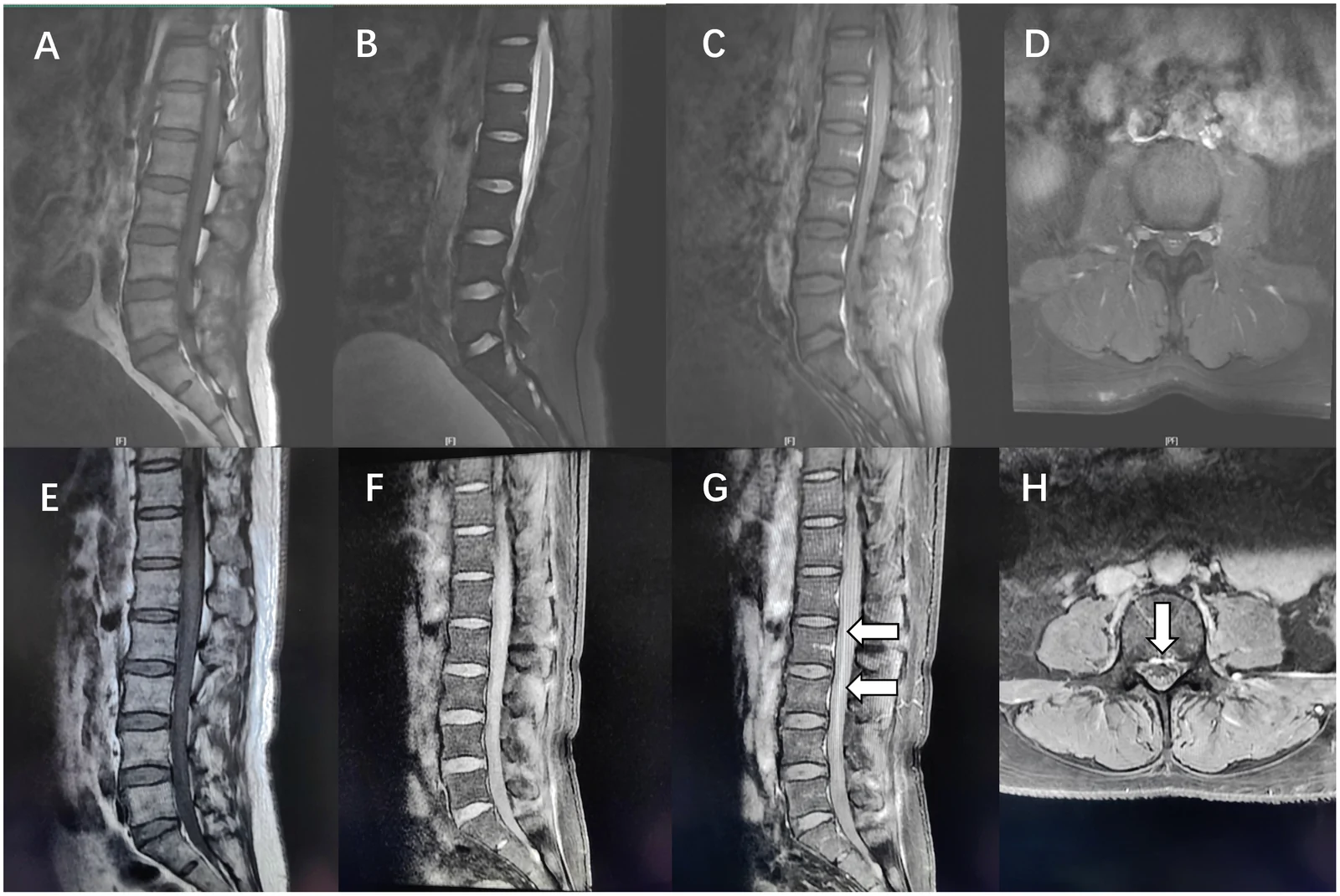
Spinal MRI findings. Initial spinal MRI on hospital day 2 **(A–D)** showed no abnormal leptomeningeal or nerve-root enhancement. Follow-up lumbar MRI about 20 days later **(E–H)** showed smooth linear enhancement along the ventral conus medullaris and cauda equina roots (arrows). This pattern supports radiculitis but is nonspecific and does not distinguish infectious meningoradiculitis from an immune polyradiculoneuropathy.

### 2.1 Diagnostic assessment, treatment, and follow-up

Electrodiagnostic testing was performed on hospital day 11 at a recorded limb temperature of 32 °C. Bilateral peroneal and tibial compound muscle action potential (CMAP) amplitudes were reduced, while distal motor latencies and conduction velocities were reported as preserved. Sural and superficial peroneal sensory responses were preserved bilaterally. F waves were not elicited; tibial H-reflex latencies were normal on both sides. Needle electromyography of the bilateral first dorsal interosseous, tibialis anterior, and medial gastrocnemius muscles showed no spontaneous activity. Motor-unit potential durations were prolonged; reported recruitment was simple. Complete nerve-level values are provided in **Supplementary Table 1**. No repeat electrodiagnostic study was performed. Several nerves also showed marked proximal-to-distal reductions in CMAP amplitude and/or area. We did not classify these changes as conduction block or reversible conduction failure because some distal CMAP amplitudes were already low and no serial study was available. GBS electrophysiological patterns can change on repeat testing, so we describe the findings as a single motor-predominant lower-limb neuropathic pattern and do not assign a definitive axonal, demyelinating, or AMAN subtype (13). No formal autonomic testing was performed; urinary retention was a clinical observation.

Lumbar puncture on hospital day 2 showed an opening pressure of 260 mmH_2_O, protein 1.56 g/L, white-cell count 272 × 10^6^/L (96.7% mononuclear), glucose 6.50 mmol/L with simultaneous serum glucose 7.6 mmol/L, and lactate 2.46 mmol/L. No CSF red cells were present, and cytology was unremarkable. CSF TPPA was reactive and CSF RPR was 1:8. Intravenous aqueous penicillin G (4 million units every 4 h) was started after the lumbar puncture and continued for 14 days. Acute myelopathy was still being considered at that point, so intravenous methylprednisolone 1,000 mg/day was also started on day 2. The dose was halved every 3 days and followed by an oral taper from 60 mg; the total corticosteroid course was 21 days. The available records did not allow a reliable cumulative methylprednisolone-equivalent dose to be calculated. Mannitol 25 g every 8 h was started for the elevated opening pressure; the stop date was not available. On hospital day 8, opening pressure was still 260 mmH_2_O. CSF protein was 1.31 g/L, white-cell count 46 × 10^6^/L, and CSF RPR 1:1. Simultaneous serum glucose was 7.2 mmol/L, no CSF red cells were present, and serum RPR remained 1:32. Both CSF RPR tests were performed in the same laboratory with the same method and technologist. The assay batch was not documented, and no stored CSF remained for parallel retesting. Analytical variability therefore cannot be excluded. Because the short-interval RPR change occurred during both penicillin and corticosteroid treatment, we did not interpret it as evidence of microbiological clearance. Neurological improvement was not yet meaningful.

Oligoclonal-band, Reiber, and neural-antibody studies used the hospital-day-8 CSF sample and serum collected the same day, after penicillin, methylprednisolone, and mannitol had been started. There were no pretreatment counterparts for these immune measures, so change from baseline cannot be assessed. Corticosteroid exposure may also have affected the day-8 inflammatory and barrier findings. Anti-ganglioside IgG/IgM testing used a qualitative line immunoblot (Attomol GmbH Molekulare Diagnostika). The laboratory record listed GM1, GM2, GM3, GM4, GD1a, GD1b, GD2, GD3, GT1a, GT1b, GQ1b, and sulfatide; MAG-IgM was also tested. Serum was diluted 1:101 and CSF 1:2. A functional line served as the run-validity control, and invalid runs were repeated. Dried strips were read visually against the batch-specific cutoff card as negative, borderline, or positive. The record did not document absolute specimen loading volumes, validated quantitative cross-matrix calibration, endpoint titers, assay lot number, or CSF-specific analytical validation. Anti-GT1a IgG was detected in CSF but not serum (**Figure 2B**); the other reported ganglioside antibodies were negative. Nodal/paranodal testing was performed by cell-based assay through Akriva Medical Laboratory Ltd using serum diluted 1:10 and undiluted CSF. NF155, NF186, NF140, CNTN1, CASPR1, and MAG-IgM were reported negative. The report did not give a numerical positivity threshold for this panel; retained-sample repeat testing was the stated quality-control procedure. Isoelectric focusing with immunofixation showed a type IV oligoclonal-band pattern with identical serum and CSF bands (**Figure 2A**). Paired CSF/serum concentrations were albumin 0.668/38.3 g/L, IgG 105 mg/L/8.92 g/L, IgA 13.6 mg/L/1.28 g/L, and IgM 17.8 mg/L/1.74 g/L. These values gave QAlb 17.4 × 10^-3^ (age-adjusted upper reference limit approximately 6.7 × 10^-3^), QIgG 11.8 × 10^-3^, QIgA 10.6 × 10^-3^, and QIgM 10.2 × 10^-3^. The IgG index was 0.67, the Reiber-derived intrathecal IgM fraction 35%, and the conventional Tourtellotte 24-h IgG synthesis rate 15.3 mg/24 h (**Figures 2C–E**). These global measures were discordant and do not resolve the source of a single antigen-specific antibody. A validated paired anti-GT1a antibody index or quantitative endpoint titers would be needed.

**Figure 2:**
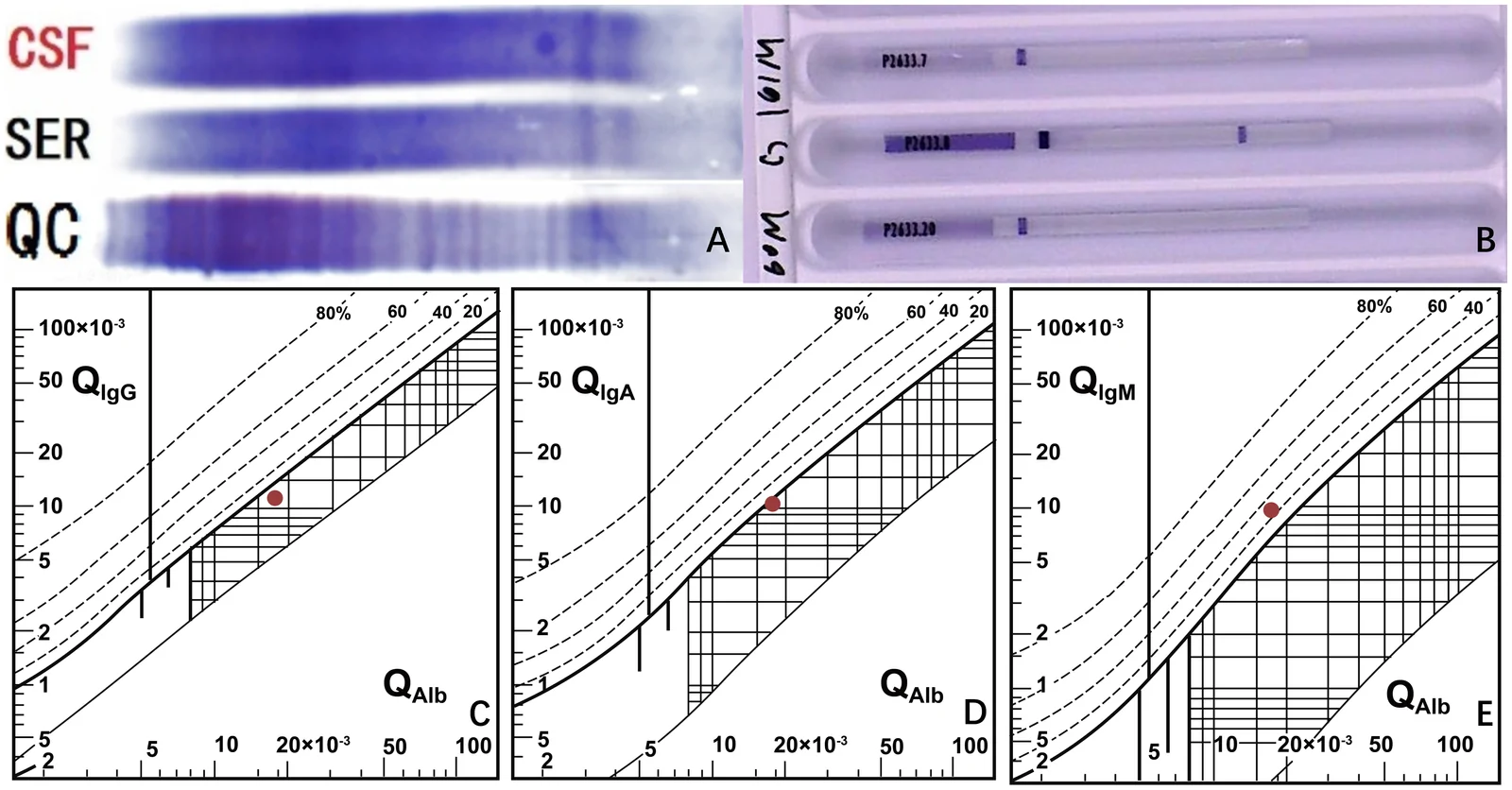
Paired CSF/serum immune assays and Reiber diagrams. **(A)** Isoelectric focusing with immunofixation showed a type IV (mirror) oligoclonal-band pattern. **(B)** A qualitative anti-ganglioside IgG/IgM line immunoblot (Attomol GmbH Molekulare Diagnostika) tested hospital-day-8 CSF and same-day serum after penicillin, methylprednisolone, and mannitol had begun. According to the laboratory record, strip antigens were GM1, GM2, GM3, GM4, GD1a, GD1b, GD2, GD3, GT1a, GT1b, GQ1b, and sulfatide. Serum was diluted 1:101 and CSF 1:2. A strip functional line was used as a run-validity control, and dried strips were read visually against the batch-specific cutoff card as negative, borderline, or positive. The supplied laboratory record did not document absolute specimen loading volumes. Anti-GT1a IgG was detected in CSF but not serum. The different dilution factors are reported descriptively. Because the assay was qualitative and no validated quantitative cross-matrix calibration, endpoint titers, lot information, or CSF-specific validation were available, the finding cannot establish relative CSF enrichment, compartmental restriction, passive transfer, or intrathecal synthesis. **(C–E)** Reiber diagrams plot immunoglobulin quotients against QAlb. Paired CSF/serum concentrations were albumin 0.668/38.3 g/L, IgG 105 mg/L/8.92 g/L, IgA 13.6 mg/L/1.28 g/L, and IgM 17.8 mg/L/1.74 g/L. QAlb was 17.4 × 10^-3^ (age-adjusted upper reference limit approximately 6.7 × 10^-3^). IgG and IgA quotients were below their hyperbolic upper discrimination limits; the IgM quotient was above its limit, corresponding to a Reiber-derived intrathecal IgM fraction of 35% (QIgM 10.2 × 10^-3^).

IVIG was started on hospital day 11 at 0.4 g/kg/day for 5 days (total dose 2 g/kg). By day 30, about 2 weeks after IVIG ended, lower-limb strength had improved to MRC 2/5, spontaneous urination had returned, and diplopia had partially improved. Formal GBS disability and modified Rankin scores were not recorded. At 2-year follow-up, the patient reported a return to normal life and work. No objective disability score, later serum or CSF RPR, repeat anti-GT1a measurement, repeat CSF assessment, or repeat electrodiagnostic study was available. We report the timing of improvement without attributing recovery to IVIG alone because penicillin, corticosteroids, rehabilitation, natural recovery, and the competing diagnosis may also have contributed. Treatment adherence, tolerability, and adverse events were not systematically documented, so we did not infer these retrospectively. **Figure 3** summarizes the clinical course, interventions, and sampling time points.

**Figure 3:**
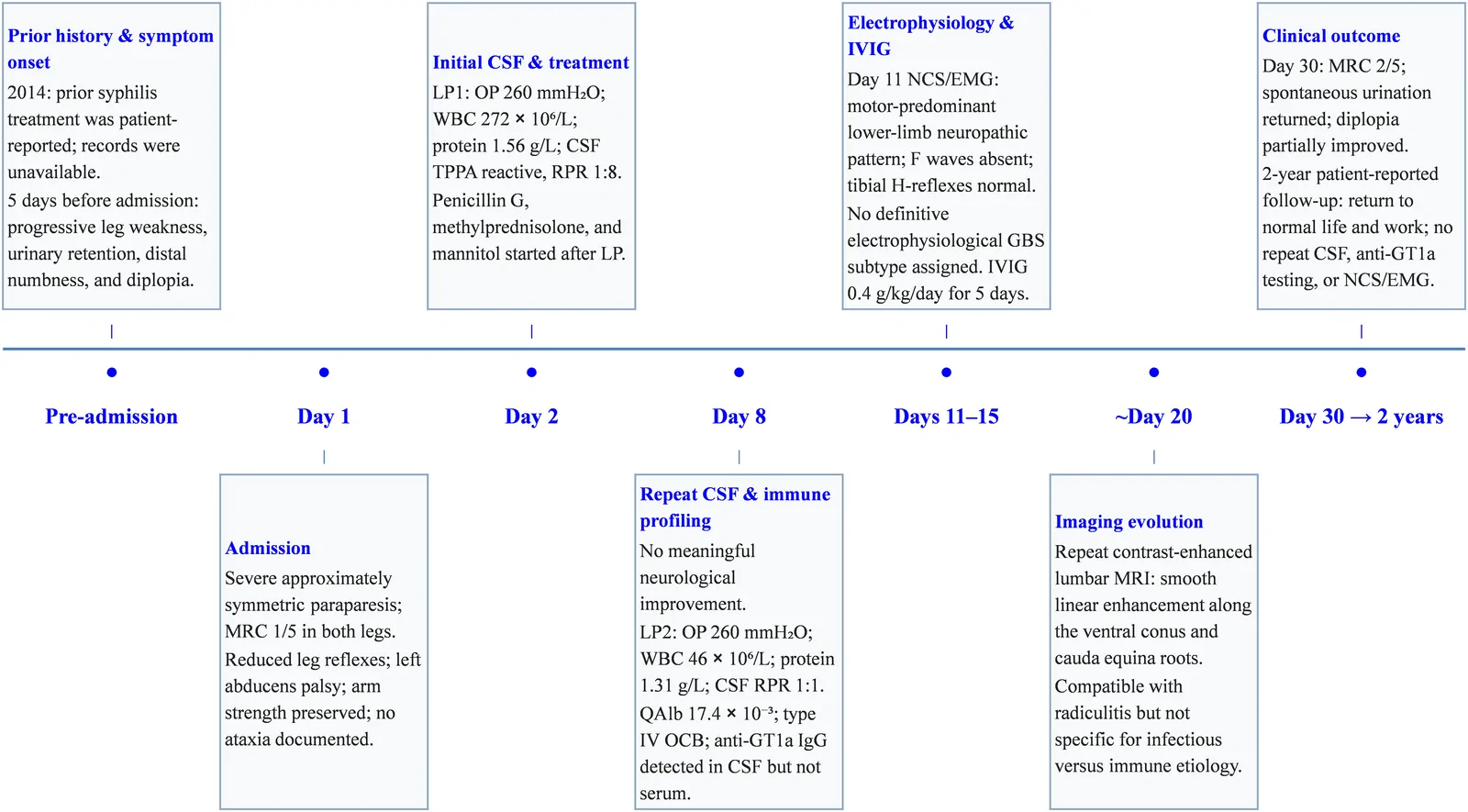
Timeline of the patient’s episode of care.

## 3 Discussion

### 3.1 Diagnostic interpretation and competing explanations

The diagnosis of active neurosyphilis is well supported by the neurological presentation, marked CSF pleocytosis, reactive CSF treponemal testing, and reactive CSF RPR. The unresolved question is whether it fully explains the acute peripheral-appearing syndrome. Rapid paraparetic weakness, leg hyporeflexia, preserved arm strength and sensory responses, reduced lower-limb motor amplitudes, and absent F waves raised the possibility of a concurrent GBS-spectrum neuropathy (1, 7, 10). However, syphilitic meningoradiculitis can itself cause polyradicular weakness, sphincter dysfunction, cranial neuropathy, CSF inflammation, and root or leptomeningeal enhancement (4, 14, 18). The available data therefore do not establish a second disease. Early CSF changes also occurred during overlapping penicillin and high-dose corticosteroid treatment, so treatment effects cannot be separated.

### 3.2 Phenotype, electrophysiology, and imaging

The weakness pattern resembled paraparetic GBS, a recognized regional GBS variant (10), but the patient did not have the classic Miller Fisher phenotype. Isolated ophthalmoparesis without ataxia or generalized areflexia is insufficient for a Miller Fisher overlap when alternative causes of cranial neuropathy remain plausible (6). Opening pressure was 260 mmH_2_O on days 2 and 8 despite mannitol, leaving a pressure-related false-localizing sixth-nerve palsy as one possibility (16). Neurosyphilitic cranial neuritis, diabetic microvascular abducens palsy, and central involvement suggested by gaze-evoked nystagmus are also possible. Anti-GT1a IgG is most often associated with cranial or oropharyngeal involvement, but absent bulbar weakness, ataxia, generalized areflexia, and anti-GQ1b antibodies limit phenotype specificity (9).

The single electrodiagnostic study showed a motor-predominant lower-limb neuropathic pattern without establishing a stable axonal or demyelinating GBS subtype. Absent F waves are compatible with proximal motor pathway dysfunction but are not specific for GBS; normal tibial H-reflex latencies are less typical in early GBS, and subtype assignment can change on serial testing (13, 19). Prolonged motor-unit durations without spontaneous activity also permit pre-existing neurogenic change. Diabetes remains a confounder, although the abrupt, approximately symmetric, painless paraparesis with HbA1c 5.5% was not typical of diabetic lumbosacral radiculoplexus neuropathy. Follow-up ventral conus-cauda equina enhancement supports radiculitis but cannot distinguish infectious from autoimmune injury (14, 15). Marked pleocytosis is a diagnostic warning sign rather than evidence for GBS (7).

### 3.3 Blood-CSF barrier dysfunction and the compartmental uncertainty of anti-GT1a IgG

QAlb was markedly elevated at 17.4 × 10^-3^, confirming substantial blood-CSF barrier dysfunction. This finding does not determine the direction of immunoglobulin trafficking or the source of an antigen-specific antibody. The type IV oligoclonal-band pattern, normal IgG index, and Reiber-based IgG analysis did not consistently demonstrate generalized intrathecal IgG synthesis; however, global IgG measures cannot determine whether one antigen-specific antibody is locally produced or differentially distributed.

Anti-GT1a IgG was detected in CSF but not serum. Serum was diluted 1:101 and CSF 1:2. These dilution factors are reported descriptively; by themselves, they do not establish analytical bias. The important limitation is that the line immunoblot was qualitative and did not provide validated quantitative calibration across serum and CSF matrices. The result therefore represents qualitative CSF-serum assay discordance rather than a quantitative comparison between compartments; it cannot establish relative antibody enrichment, compartmental restriction, or intrathecal synthesis. Low-level systemic antibody below the serum detection threshold, matrix-dependent analytical sensitivity, altered compartmental distribution, selective retention, and local synthesis remain possible and cannot be distinguished with the available assay. Samples were obtained after treatment had begun, adding further uncertainty. The elevated Tourtellotte IgG synthesis rate (15.3 mg/24 h) was discordant with the IgG index, type IV OCB pattern, and Reiber QIgG in the setting of marked barrier dysfunction (11). The Reiber-derived intrathecal IgM fraction of 35% supports compartmental IgM activity but does not establish intrathecal anti-GT1a IgG synthesis or a treponemal cause. We therefore treat anti-GT1a as an immune finding of uncertain pathogenic significance rather than as a diagnostic marker. Quantitative paired anti-GT1a measurements using a method validated for both matrices, endpoint titers or a validated antigen-specific antibody index, and longitudinal paired samples would be required to resolve its origin.

### 3.4 A testable “two-hit” framework for infection-autoimmunity convergence

These observations can be integrated into a modified “two-hit” framework, not as a demonstrated causal sequence but as a model that can be tested. The first “hit” is active treponemal neuroinflammation with marked blood-CSF barrier dysfunction and meningoradicular inflammation, potentially creating a permissive neuroimmune environment. The second is an antiganglioside immune signal occurring during an acute motor-predominant polyradicular syndrome. The model does not assume that circulating anti-GT1a crossed into CSF, nor does it establish local synthesis or antibody pathogenicity.

Within this framework, the delayed ventral conus-cauda equina enhancement may mark an anatomical site where infectious meningoradicular inflammation and a putative immune root process overlap, but MRI cannot identify the mechanism (14, 15). Three explanations remain possible: syphilitic meningoradiculitis alone; neurosyphilis with an independent GBS-spectrum neuropathy; or a biologically linked infection-autoimmunity process. Molecular mimicry is a recognized mechanism in GBS (12), and bystander immune activation is another theoretical possibility, but neither was tested in this patient. The framework is useful because it can be tested. Paired quantitative anti-GT1a measurements or an antibody index, serial QAlb, repeat electrophysiology, and longitudinal CSF and imaging could show whether immune, barrier, anatomical, and neurological changes move together over time. If these measures changed in parallel with the clinical syndrome, that would support a linked process; if they changed independently, it would argue against it. The six-day CSF RPR decline is too short to establish microbiological clearance (17). Improvement after IVIG remains a temporal association in this uncontrolled case; penicillin, corticosteroids, rehabilitation, and natural recovery may also have contributed (12).

**Table 1** places the present case alongside three published neurosyphilis reports with GBS- or Miller Fisher-like presentations (2, 3, 8). Across these reports, infectious and immune-neuropathy phenotypes overlapped, and immunotherapy was given during antitreponemal treatment or before neurosyphilis was recognized. They support considering competing or coexisting processes but do not establish a shared mechanism. Proven neurosyphilis requires prompt treatment (4). If a concurrent GBS-spectrum neuropathy remains clinically likely and disability is severe, IVIG or plasma exchange may be considered while infectious and structural causes are addressed (1, 7, 12). In this case, treatment choice and later improvement cannot serve as retrospective diagnostic proof.

**Table 1:** Published neurosyphilis cases with Guillain-Barré- or Miller Fisher-like presentations.

Report	Clinical phenotype	CSF/electrophysiology	Treatment and interpretive limitation
Berger et al. (2)	51-year-old man; ascending sensory and motor symptoms	CSF WBC 73/µL, protein 148 mg/dL, VDRL 1:2; electrophysiological abnormalities interpreted as GBS	Penicillin plus IVIG; improvement reported; diabetes and recent COVID-19 vaccination were potential confounders
Ung and Ferrey (3)	37-year-old woman; weakness, dysphagia, multiple cranial neuropathies	Reactive CSF VDRL with only slight pleocytosis; mixed demyelinating polyneuropathy predominantly affecting motor nerves	Penicillin plus plasma exchange; near-complete recovery by discharge reported
Stepper et al. (8)	36-year-old man; diplopia, severe ataxia, areflexia, sixth-nerve palsy	CSF pleocytosis with protein 2.4 g/L; initial presentation mimicked Miller Fisher syndrome	Initially treated as Miller Fisher syndrome; sixth-nerve palsy improved after IVIG but later relapsed; active neurosyphilis was subsequently recognized and treated with penicillin
Present case	41-year-old man; paraparetic weakness, urinary retention, sixth-nerve palsy without ataxia	CSF WBC 272 × 10^6^/L, protein 1.56 g/L, RPR 1:8; single day-11 study at 32 °C with reduced motor amplitudes, preserved sensory responses, absent F waves, and normal tibial H-reflex latencies; anti-GT1a IgG detected in CSF but not serum by a qualitative line blot without validated quantitative cross-matrix calibration	Penicillin, methylprednisolone, mannitol, then IVIG; delayed improvement; no serial NCS, quantitative antibody testing, or post-treatment CSF; syphilitic meningoradiculitis, multiple treatments, assay design, diabetes, and natural history remain confounders

### 3.5 Strengths and limitations

Several features strengthen this report: serial CSF measurements, same-day paired serum-CSF immune testing, nerve-level electrodiagnostic data with testing conditions, sequential spinal imaging, an episode-of-care timeline, and long patient-reported follow-up. The principal limitations concern diagnostic attribution, mechanistic inference, and treatment causality. A single electrodiagnostic study cannot establish a stable GBS subtype, and marked pleocytosis plus syphilitic meningoradiculitis preclude confident retrospective Brighton classification. For anti-GT1a, the principal analytical limitation is that the assay was qualitative and lacked validated quantitative cross-matrix calibration, endpoint titers, and an antigen-specific antibody index; the different serum and CSF dilution factors are reported descriptively rather than treated as a limitation in themselves. Antibody origin and pathogenicity therefore remain unresolved, and immune testing occurred after treatment began. Penicillin, corticosteroids, mannitol, IVIG, rehabilitation, and natural recovery overlapped, preventing attribution of improvement to a single intervention. Follow-up CSF, serial RPR, repeat anti-GT1a testing, repeat electrophysiology, formal autonomic testing, and prospective disability scores were unavailable. The 2014 syphilis treatment history was patient-reported, so relapse or treatment failure cannot be distinguished from reinfection. These limitations define the interpretive boundary: the case supports a structured, testable hypothesis, not proof of dual pathology or an infection-autoimmunity causal sequence.

### 3.6 Conclusion

This case illustrates the difficulty of separating syphilitic meningoradiculitis from a concurrent immune polyradiculoneuropathy when active neurosyphilis, a paraparetic GBS-spectrum phenotype, marked blood-CSF barrier dysfunction, and CSF-detected anti-GT1a IgG occur together. The CSF-serum anti-GT1a discordance is an immune finding of uncertain pathogenic significance and is not diagnostic. A modified two-hit framework can organize the competing explanations, but testing it requires quantitative paired serum-CSF antibody studies, serial barrier and immune measurements, repeat electrophysiology, and longitudinal imaging.

## 4 Patient perspective

The patient provided the following statement regarding his illness and recovery: “When the weakness rapidly paralyzed my legs and I lost the ability to urinate, I felt completely terrified and helpless. However, shortly after receiving the intravenous immunoglobulin treatment, I could feel my strength gradually returning, which gave me tremendous hope. Now, two years later, I have fully returned to my normal life and work, and I am deeply grateful to the medical team for their accurate diagnosis and care.” This personal account is presented separately from the authors’ causal interpretation.

## Data Availability

The original contributions presented in the study are included in the article/**Supplementary Material**. Further inquiries can be directed to the corresponding author.
