# Supplementary material for: Case Report: Neurosyphilis with a paraparetic Guillain-Barré-spectrum phenotype and CSF-detected anti-GT1a IgG: an infection-autoimmunity diagnostic challenge
Source: Front Immunol. 2026 Sep 14;17:1936622. doi: 10.3389/fimmu.2026.1936622 (PMC13617122; doi:10.3389/fimmu.2026.1936622)
Supplement: Supplementary file 1 [file Table1.docx]

**Supplementary Table S1. Complete available electrodiagnostic data**

The examination was performed on hospital day 11 at a recorded limb temperature of 32°C. F waves were not elicited; no repeat electrodiagnostic study was performed.

**A. Needle electromyography**

| **Muscle** | **Spontaneous activity** | **Mean MUP duration (ms)** | **Polyphasic MUPs (%)** | **MUP amplitude (mV)** | **Recruitment/interference pattern** |
| --- | --- | --- | --- | --- | --- |
| Right first dorsal interosseous | None | 14.3 | 0 | 3.34 | Simple |
| Left first dorsal interosseous | None | 16.2 | 100 | 0.97 | Simple |
| Right tibialis anterior | None | 16.6 | 67 | 0.91 | Simple |
| Left tibialis anterior | None | 17.0 | 50 | 0.45 | Not stated |
| Right medial gastrocnemius | None | 16.5 | 0 | 0.56 | Simple |
| Left medial gastrocnemius | None | 16.3 | 50 | 1.33 | Simple |

**B. Motor nerve conduction**

| **Nerve** | **Stimulation segment/site** | **Latency (ms)** | **Duration (ms)** | **CMAP (mV)** | **Area (mV·ms)** | **MCV (m/s)** | **Duration diff. (%)** | **Amplitude diff. (%)** | **Area diff. (%)** |
| --- | --- | --- | --- | --- | --- | --- | --- | --- | --- |
| Right common peroneal | Ankle | 4.60 | 8.80 | 2.13 | 7.92 | — | — | — | — |
| Right common peroneal | Fibular head–ankle | 13.9 | 6.80 | 1.20 | 2.97 | 38.7 | -22.7 | -43.6 | -62.5 |
| Left common peroneal | Ankle | 4.45 | 16.8 | 0.70 | 7.67 | — | — | — | — |
| Left common peroneal | Fibular head–ankle | 13.0 | 21.3 | 0.12 | 0.58 | 41.9 | 26.4 | -83.3 | -92.4 |
| Right tibial | Medial malleolus | 4.60 | 7.20 | 1.35 | 2.81 | — | — | — | — |
| Right tibial | Popliteal fossa–medial malleolus | 11.5 | 8.50 | 0.55 | 1.58 | 46.4 | 18.1 | -58.9 | -43.8 |
| Left tibial | Medial malleolus | 4.15 | 9.45 | 1.08 | 4.04 | — | — | — | — |
| Left tibial | Popliteal fossa–medial malleolus | 12.1 | 9.40 | 0.92 | 1.95 | 40.0 | -1.58 | -14.8 | -51.8 |

**C. Sensory nerve conduction**

| **Nerve** | **Stimulation site** | **Recording site** | **Latency (ms)** | **Duration (ms)** | **SNAP (µV)** | **Area (µV·ms)** | **Distance (mm)** | **SCV (m/s)** |
| --- | --- | --- | --- | --- | --- | --- | --- | --- |
| Right sural | Above heel | Lateral malleolus | 2.23 | 1.57 | 17.2 | 14.5 | 90 | 40.3 |
| Left sural | Above heel | Lateral malleolus | 2.20 | 2.17 | 12.2 | 14.6 | 90 | 40.9 |
| Right superficial peroneal | Peroneus longus | Dorsum of foot | 3.47 | 1.56 | 14.8 | 11.1 | 140 | 40.4 |
| Left superficial peroneal | Peroneus longus | Dorsum of foot | 3.50 | 1.17 | 11.6 | 4.56 | 150 | 42.9 |

**D. H-reflex**

| **Nerve** | **Stimulation site** | **M latency (ms)** | **H latency (ms)** | **H–M latency (ms)** | **M amplitude (mV)** | **H amplitude (mV)** | **H/M amplitude ratio** |
| --- | --- | --- | --- | --- | --- | --- | --- |
| Right tibial | Popliteal fossa | 4.67 | 32.8 | 28.1 | 0.57 | 0.05 | 0.08 |
| Left tibial | Popliteal fossa | 4.58 | 32.8 | 28.2 | 0.74 | 0.33 | 0.44 |

Original report conclusion: distal motor latencies and motor conduction velocities in the bilateral common peroneal and tibial nerves were reported as normal, with reduced CMAP amplitudes; bilateral sural and superficial peroneal SNAP amplitudes and sensory conduction velocities were reported as normal; bilateral tibial H-reflex latencies were normal; and F waves were not elicited. In the present manuscript, these findings are described conservatively as a motor-predominant lower-limb neuropathic pattern. A definitive AMAN, AIDP, or other stable GBS electrophysiological subtype cannot be assigned from a single study, particularly in the presence of infectious meningoradiculitis and diabetes-related neuropathic confounding.
